# Sudan’s Endless Exodus and the Cruel Geometry of the Refugee Loop

**DOI:** 10.7759/cureus.95433

**Published:** 2025-10-26

**Authors:** Muhammad Hamza Shah, Leena Ahmed Osman

**Affiliations:** 1 School of Medicine, Queen's University Belfast, Belfast, GBR; 2 Department of Internal Medicine, Antrim Area Hospital, Antrim, GBR; 3 Centre for Anatomy, The University of Edinburgh, Edinburgh, GBR; 4 Department of Paediatrics, Antrim Area Hospital, Antrim, GBR

**Keywords:** displacement, humanitarian crisis, internal displacement, refugee loop, sudan

## Abstract

Sudan faces the world’s largest displacement crisis. Since the war began in April 2023 between the Sudanese Armed Forces and the Rapid Support Forces, more than 12 million people have been forced from their homes. This movement of people, described as Sudan’s 'refugee loop', has created a pattern of flight, temporary refuge, and unsafe return that reveals how violence and neglect have become deeply organised into daily life. Camps inside and outside Sudan now mark the geography of this collapse, where famine, disease, and the deaths of children unfold with little international attention. Hospitals lie in ruins, schools remain closed, and farmlands are barren. The article argues that this crisis is no longer only humanitarian. It has become a political arrangement in which displacement itself holds the country together, sustaining power through abandonment and silence. Breaking Sudan’s displacement cycle means restoring the conditions that let people live and stay. That begins with political commitment that lasts beyond headlines, and ceasefires that are enforced rather than declared. Neighbouring states carrying the weight of Sudan’s refugees also need real help to keep their systems from breaking. Without these efforts, displacement will harden into a permanent way of life. The real measure of progress is whether people can return home and find more than ruins waiting for them.

## Editorial

Sudan is living through the world’s largest displacement crisis. Since the war erupted in April 2023 between the Sudanese Armed Forces and the Rapid Support Forces, more than 12 million people have been forced from their homes, including 7.7 million internally displaced persons (IDPs) and over four million who have crossed borders into neighbouring countries, with more than half of these being children [1]. For many, this is not their first journey into exile, it is the latest turn in a relentless cycle: flee violence, seek refuge in a camp or across a border, return in hope of rebuilding, and then flee again when the next wave of conflict arrives. This is Sudan’s 'refugee loop' - a cruel rhythm in which people are constantly on the move yet never able to arrive [2]. The loop is driven by three overlapping crises: unending violence, the collapse of basic systems, and the indifference of the international community. In South Sudan, thousands of returnees who once sought refuge there are now stuck in overcrowded camps where food is scarce, healthcare minimal, and security fragile. Some attempt to return to Sudan, only to find famine advancing and farmland either burned or mined.

Refugee camps inside Sudan are no safer. Zamzam camp, now sheltering around half a million people, is gripped by severe malnutrition, and at least one child dies every two hours. In August 2025, a Rapid Support Forces assault on Zamzam killed between 1,500 and 2,000 people in one of the gravest atrocities of the war [3]. In Abu Shouk camp, a similar attack killed at least 40 people, while malnutrition claimed 60 lives in a single week [4]. Camps that were once meant to be sanctuaries have become new frontlines. The structural collapse is staggering. Over 70% of hospitals in conflict zones and almost half in other areas are non-functional, and more than 25 million Sudanese, over half the population, are facing acute food insecurity [5]. The education system is in ruins. For displaced children, schooling has been interrupted for years; many will never return to class. Malnutrition is widespread, with long-term effects on physical and cognitive development. The next generation is being shaped in conditions that all but guarantee a fragile future.

Consequently, one cannot term it as a humanitarian crisis - it is a failure of political will. The collapse of peace talks is not simply a matter of mistrust between warring factions; it reflects a deliberate strategy by elites who benefit from perpetual conflict, both in Sudan and in neighbouring states that see instability as leverage. Regional powers with stakes in Sudan’s war have prolonged the conflict through arms supplies and proxy alliances, turning Sudan into yet another arena of geopolitical rivalry [6]. Meanwhile, international attention has shifted elsewhere, to Ukraine, Gaza, and other high-profile conflicts, leaving Sudan dangerously underfunded and treated as peripheral. The result is an 'invisible emergency', where humanitarian operations are forced to ration aid, even as needs escalate beyond measure. However, to reduce Sudan’s crisis to aid gaps misses the structural dimension. Breaking Sudan’s refugee loop requires dismantling the very political economy of displacement. Displacement in Sudan has become a mechanism of control, a way to reorder land, demographics, and access to resources. Violence against cities like El-Fasher shows how siege, shelling, and drone strikes forcibly reshape population movements [7]. Justice must therefore reach beyond field commanders to financiers, smuggling networks, and cross-border enablers. Without addressing this, humanitarian interventions risk perpetuating cycles rather than breaking them. Long-term strategies must therefore go beyond stabilising communities - they must safeguard displaced people’s political rights, land ownership, and access to justice, so that return is not only safe but also meaningful.

This also means moving beyond 'event-based' donor responses. Every flare-up in Sudan is met with short-lived pledges that evaporate when media cameras turn away. What is needed instead is a model of sustained, multi-year investment tied to reconstruction benchmarks, where donors are held accountable for continuity, not applause-driven announcements. Ceasefire agreements too must not remain symbolic. They require enforcement mechanisms credible enough to deter violations with sanctions targeting financiers of war economies, arms embargoes monitored with technological rigor, and clear regional accountability frameworks that prevent neighbouring states from quietly profiting from Sudan’s misery [8].

Infrastructure cannot wait for peace to magically 'settle'. Hospitals must be restored, schools reopened, farmland cleared of landmines, and water networks rebuilt so that returning home does not mean returning to ruin. Economic revival is not a postscript but a precondition of peace. The reconstruction of markets and agricultural systems would allow displaced people to rebuild livelihoods, reducing dependence on aid and undercutting the appeal of armed recruitment. Equally, host countries like Chad, Egypt, Ethiopia, and South Sudan cannot be left as overstretched buffers. Their fragile systems are buckling under the weight of refugee inflows, producing resentment that fuels xenophobia and premature, unsafe returns. Supporting these states with debt relief, infrastructure funding, and inclusive refugee policies is therefore not charity but prevention - an investment in avoiding future conflict spillovers [9]. Accountability must also be understood more broadly than punishing commanders after atrocities. Impunity has kept Sudan’s wars on a perpetual loop; justice could help break it. But justice must encompass the financial and logistical enablers of war - arms dealers, corrupt banking networks, and foreign backers who fuel impunity from afar. Targeted sanctions, asset freezes, and transparent investigations into resource smuggling (gold, oil, and arms) would cut the economic arteries of war. Without this, international courts risk trying individuals while leaving the war economy intact.

Above all, Sudan’s displacement crisis must be brought back into the global conscience. This should not be seen as a peripheral emergency in Africa - it is a central test of whether the international community is willing to confront protracted displacement as a systemic failure rather than an unfortunate side-effect of war. Sudan’s refugee loop will not break itself. Without political courage, adequate resources, and a commitment to rebuilding lives as well as infrastructure, every return will lead to another departure, and every fragile homecoming will dissolve into another forced flight. The measure of success, therefore, is not the number of tents erected or meals distributed this month, but whether those displaced can go home and stay there. Until that is possible, Sudan will remain a nation in motion, a living reminder that wars do not simply destroy homes - they destroy the very idea of home. Unless the world treats Sudan not as a distant humanitarian problem but as a mirror of its own failure to uphold the most basic covenant of human security, the cycle of displacement will remain unbroken, and with it, the erosion of global norms that claim to protect the vulnerable [10].
